# Sugar-Sweetened Beverages: General and Oral Health Hazards in Children and Adolescents

**DOI:** 10.5005/jp-journals-10005-1094

**Published:** 2010-04-15

**Authors:** MB Mishra, Shanu Mishra

**Affiliations:** 1 Former Associate Professor, School of Dentistry, Loma Linda University, Loma Linda, California, USA; 2 Registrar, Rotherham General Hospital, Rotherham, Yorkshire, United Kingdom

**Keywords:** Sugar-sweetened beverages (SSB), Type-2 diabetes, Dental caries, Dental erosion, Body mass index (BMI).

## Abstract

Ubiquitously unhealthy eating and drinking habits and the development of multiple morbidities, including obesity, type-2 diabetes, dental caries and dental erosion have become a major challenge for physicians, dentists and parents. Modernization has provided heaps of option for outdoor eating and sugar-containing drinks. Even the “diet” labeled drinks are considered not free from sugars and enhances calorie input. With the increasing trends of eating unhealthy, sticky and readily available, refined carbohydrate-rich foods and drinks, problems pertaining to body’s metabolic activity and oral health have also been significantly recognized. Dentists and pediatricians can play a pivotal role and should emphasize on patients’ education and counseling on the proper nutritional diet and health.

## INTRODUCTION

With the substantive increase in oral hygiene awareness, use of fluoridated toothpastes and visiting a dental office has reduced the tooth decay (dental caries) by 30%. Children, young adults, elderly and community living below poverty level have more problems with dental caries.^1^

Dental caries is a multifactorial chronic disease and its severity is influenced by factors, such as bacteria, saliva, sugar intake, oral hygiene, diet, fluoride exposer and genetics. In developing countries, the dentists have experienced significant decrease in dental caries in last 30 years because of dental health awareness, water fluoridation, pit and fissure sealants, fluoridated tooth pastes, etc. But still rampant caries remains a problem in the specific groups: children, young adults, elderly people and those living below poverty level. Most dentists agree that diligent home care, fluoride application, healthier eating habits and limiting sugar intake are essential ailments for good general and oral health for almost every individual.

Patient’s teeth restorations done by a dentist, provides a preventive oral health plan and scheduling the patients for regular oral check-ups. Simultaneously, dentist should educate their patients on the impact of diet on the progression of dental caries and overall health. This article emphasizes on unhealthy eating and drinking habits and the development of multiple morbidities, including obesity, type-2 diabetes, caries and dental erosion, and further should stress on patients’ education and counseling on the diet and health.

### Dental Caries―Let the Mothers Understand

Educate your patients and parents that dental caries is one of the most common of all diseases and is preventable, transmissible, infectious disease. The mothers should know that bacteria of their mouth are transmitted to their infants. If the mother has active caries, then it is likely that she can transfer cariogenic bacteria to their babies’ teeth.^2^

Teaching a patient or parents should include diet counseling and the impact that frequency of sugar consumption on dental caries risk. Fluoride regimens, proper brushing, flossing and regular check-up are best recommended preventive measures.

It has ubiquitously been observed and accepted that mechanical cleansing method (adequate brushing) is most potential measure in preventing microbial plaque from the teeth, rest other measures used are adjuncts. Brushing at home should be taken care by the parents for the toddlers and young children to minimize dental caries risk and restrain further damage.

### Frequency of Sugar Consumption

Clear and extensive evidence has been established to prove correlation between the frequency and amount of sugars containing products and prevalence and severity of dental caries and erosion.

The frequency of consumption is crucial. “Snaking or grazing” results in plaque pH being below the point where net outflow of calcium and phosphate ions from tooth surface occurs for prolonged periods.

Carbonated drinks have a pH of 2 to 3 and can cause marked loss of tooth structure via erosion, an increasing problem in teenagers. Literature also suggests that even diet varieties can lead to erosion.

To discourage sugars in sugar-sweetned beverages (SSB), nonsugar sweeteners (NSS) are to be encouraged because of their group property since they are noncariogenic and useful sugar substitutes. These (NSS) are:

1. Bulk sweeteners, e.g. sorbitol and xylitol, provide calories and bulk, useful substitute in confectionary, chewing gum and medicines.
2. Intense sweeteners, e.g. saccharin and aspartame, are calorie free, popularly known as slimmer foods.

Above both types, from dental point of view, whilst bulk and intense sweeteners are noncariogenic, and therefore useful sugar substitutes. Use of artificial sweeteners perpetuates the craving for sweet foods. The factors related to changing behavior are particularly important in encouraging patients to adopt a less cariogenic diet.

Effective dietary counseling requires knowledge of patients’ dietary habits related to nonmilk extrinsic sugar consumption. Sugar-free chewing gums stimulate saliva and thus increases salivary buffers and enhances washout of sugars. Chewing gums should be advised as prime caries and bacterial plaque preventive measure. Sugars are generally and broadly classified on the basis of biochemical or structural character like, polysaccharide, disaccharides, monosaccharide, oligosaccharides, etc.

For practical purposes, another classification used is as follows:

**Table:** 

*Intrinsic sugars*	
• Sugars forming an integral part of certain unprocessed foodstuff are called intrinsic because they are enclosed within a cell	
• Found in whole fruits and vegetables; mainly fructose, glucose and sucrose.	
*Extrinsic sugars*	
• Found in food outside the cellular structure. They are further classified:	
– Milk sugars―in milk and milk containing products mainly lactose.	
– Nonmilk extrinsic sugars―found in confectionary, soft drinks, cakes, biscuits, etc. including sucrose, fructose and glucose.	
• Comprises two-third of all sugars and have greatest cariogenic potential.	

Evidence in the literature is found stating an established relationship between frequency of carbohydrate intake and caries activity. More frequent exposure to sugar between meals increased caries activity and higher decayed, missing, filled (DMF) index, compared to sugar consumption at meals. Four and more soft drinks between meals are associated with 179% increase in odds of having a high DMF.^3^

Other reports have shown that high sugar intake, dietary sequence, frequency and form of carbohydrate intake have important role in dental caries expression.^4,5^ Confectionary, soft drinks, biscuits and cakes contain nonmilk extrinsic sugars (NMES), including sucrose, fructose and glucose. NMES comprise two-thirds of all sugars in diet and have the greatest cariogenic potential.

### Effect of SSB on BMI and Bones

In last 50 years the consumption of soft drinks has increased by 500 times and the consumers are predominantly children, teens, and young adults and mainly in summer.^6^ Consumption of soft drinks has been found 73% in boys and 62% in adolescent girls.^7^

American Academy of Pediatrics has recommended reduction of soft drink consumption and banning sale of soft drinks in school vending machines, because, these soft drinks are the leading source of added sugar in the diet of adolescents.^8^ Association has been established by several studies between sugar-sweetened beverages and the incidence of obesity. Children taking highest level of sugar-sweetened beverages had gained weight.

Current evidence strongly suggests that sugar-sweetened beverages (SSB) intake is a marker of unhealthy lifestyle and increase the risk of obesity and type-2 diabetes.^9^ Several studies clearly suggest that one of the consequences of excessive soda drinking is excessive caloric intake.

An 8-year prospective study of 91,249 nurses demonstrated a significant association between excessive caloric intake from soda and both the development of obesity and type-2 diabetes.^10^ There is an increased trend among children, adolescent and young boys and girls for consumption of high caloric-density foods, meals, snacks and sweetened beverages away from home and with relatively no physical activity. Soft drinks have harmful effects since they are potential source of sugar, caffeine, acid and calories, and their subsequent effects on weight, bones and teeth.

It has been proved that children and adolescents who skip breakfast have higher body-mass-index (BMI), because skipping breakfast may lead to imbalance eating later in the day by consumption of SSB, snacks and calorie food. 20 oz can of soda contains 16 TSF of sugar, 250 calories, 90 mg of caffeine, addition to it, the pH of 3 or lower, and contains no nutrients, vitamins or calcium. For the development of healthy bones and teeth, calcium is very essential in children and adolescents.

Milk is the principle source of calcium and vitamin D. By 18 years of age, women accumulate their skeletal bone mass and density.^11^ Peak bone mass is key determinant of osteoporosis in later life.

Diminished calcium intake jeopardizes bone density and significantly increases the risk of osteoporosis. Wyshak in his research has established high correlation between bone fracture and carbonated cola beverages intake among physically active girls.^12^ It is suspected that the phosphoric acid in cola drinks may chelate dietary calcium, preventing gastrointestinal absorption. Soda and beverages have replaced milk of the diet of children. Calories added to the typical diet with no increase in physical activity can lead to a weight gain of 15 lb per year with a single 12 oz can consumption daily.^9^

The prevalence of diabetes worldwide is increasing rapidly in association with the increase in obesity. Regular and enormous consumers of SBB are obese and have increased percentage of body fat distributed predominantly in the abdominal region. The normal body mass index (BMI) in a young adult is under 25 kg/m^2^, whereas BMI between 25 and 30 kg/m^2^ is defined as overweight, the BMI over 30 kg/m^2^ is defined as obese. It has been suggested that adipose tissue plays an important role in development of insulin resistance. Increased level of free fatty acid derived from adipocytes; contribute in insulin resistance by inhibiting glucose uptake, glycogen synthesis, glycolysis, and by increasing hepatic glucose production. Weight reduction can improve insulin resistance.

Complications are major fear with diabetes. Complications of diabetes affect many tissues and organs, causing retinopathy, neuropathy, nephropathy, cardiovascular diseases, stroke and periodontal pathogenesis. Immunological abnormalities are associated with type-1 and type-2 diabetes and diabetic complications.

In type-2 diabetes, inflammation and activation of mono-cytes are postulated to be important for enhancing insulin resistant and may contribute to the loss of insulin secretory function of islet cells. Among the other contributory factors, diet, sedentary life style and obesity greatly contribute to enhance insulin resistance.^13^

Abnormal level of metabolites, such as lipids, fatty acids, and various cytokines from adipose tissue, activate adipose tissue, and monocytes, and increase the level of cytokines, enhances insulin resistance. George L King (2008) has mentioned that obesity activates monocytes and enhances insulin resistance, thereby increasing risk of diabetes type-2.^14^

Incidence of diabetes is increasing rapidly and predicted to increase further in parallel with trends observed for obesity.^15,16^

According to Center for Diseases Control (CDC) report (2005), the prevalence of type-2 diabetes is increasing in adolescents in conjunction with childhood obesity.^17^

Dr Robert Genco suggested that the increased risk for periodontal disease exists in patients with obesity without diabetes. His findings suggest that periodontal disease associated with diabetes may be more related to macrovascular complications of diabetes.

Amos AF et al (1997) in their study projected fear of increased people with diabetes worldwide to reach 221 million and in certain regions like Asia and Africa, diabetes rates could be two-fold or three-fold.^18^

### Cariogenicity and pH-Induced Tooth Erosion

Multiple factors play significant role in causing caries of teeth like:

- Food form and frequency of consumption
- Sequence
- Fluorides
- Combination of other foods.

Cariogenicity of sugary foods and drinks has been found higher when consumed between the meals as compared with when consumed during the meals.

Zero in 2004, stated that “fluoride has raised the threshold of sugar intake at which caries will progress to cavitations, but fluoride has its limits and caries remain a serious problem for disadvantaged individuals in many industrialized countries.^19^

Populations at large who consume these kinds of beverages do not consider the risk and are unaware of their potential damage to the teeth. Most commercial beverages are acidic and their pH range from 2.5 to 3.3.^20^

Damage (erosion) to the tooth enamel and dentine occurs and the percentage of tooth material loss increases with exposer time. One sip all day should be made aware of risks of prolonged exposer of teeth to acidic beverages.

The optimal neutral pH of oral cavity is 6.75 to 7.25, the threshold pH for dental caries development is 5.5 and dentine erosion occurs at a pH of 6.0.

After sugar consumption, the pH in plaque can fall rapidly to below 5.0 by the production of acids (predominantly lactic acid) by bacterial metabolism. Many of the predominant bacterial components of dental plaque associated with healthy sites can tolerate this fall in pH.

These conditions are likely to occur in subjects who commonly consume sugar-containing snacks, or drinks between meals. This can result in the enhanced growth of or colonization by acid―tolerant (aciduric) species, such as *streptococcus mutans* (and related species) and *lacto-bacillus species,* which are normally absent or only minor components found in dental plaque. Such a change in the bacterial composition of plaque predisposes a surface to dental caries.

Teeth erosive potential of these beverages depends on certain other factors, such as, composition, retention in oral cavity, type and strength of acid(s), pH value, total acid level, calcium chelating properties, calcium and phosphate contents and buffering capacity.

The patient factors contributing in erosion are:

- Frequency of consumption
- Method of drinking
- Fluoride exposer
- Salivary pellicle
- Salivary buffering capacity
- Salivary flow rate.

Noncola drinks, commercial lemonades, energy and sports drinks exhibited more aggressive dissolution of enamel (p < 0.05) than black tea and cola drinks.^21^ It has been suggested that erosive potential can be reduced by adding calcium and phosphate to the beverages.^21^

Now there is turning trend among children and adults to eat meals away from home and consume fast food and soft drinks bundled conveniently in an attractive packaging and price. A recent intervention program has shown that a reduction in SSB consumption resulted in significant weight loss among adolescents.^22^

### Eating Pattern and Family Impact

Since childhood the pattern of eating develops and can influence long-term nutritional status. Many studies have established relationship of food and beverages and weight gain, obesity and diminished intake of nutrients. Research by Duffey and Popkin suggested that food and beverage intake are linked intimately and that persons with healthier dietary pattern had healthier beverage consumption.^23^

Health education involves the provision of information aimed at influencing beliefs, attitudes and behavior relating to general, oral and dental health. The key messages are:

- Reduce sugar containing food and drinks, particularly frequency of sugar containing drink consumption. Avoid sugar snacks between meals.
- Parents may realize that fresh fruit is preferable to SSB, chocolate bars, nonavailability or price may preclude its provision.
- Similarly SSB or sugar containing foodstuffs are given to children not only when they are hungry but also as a reward or a pacifier.
- Brushing teeth daily with toothpaste containing fluoride
- Attend a specialist dentist regularly
- On been observed a child overweight or unproportionate body growth to chronological age, must consult physician for further prevention and needful measures.

There should be provision of appropriate information and circumstances, belief and attitudes of individuals that will be affected sufficiently to result in the adoption of behavior likely to enhance health and diminish the chance of disease.

### Advice to Patients and Parents

Dentist should advise and encourage the patients and parents for limiting their consumption to few commercial beverages. Also discourage screw-topped bottles and recommend a canned drink. A screw-top bottle preserves carbonation, allows recapping and repeated sipping, whereas in a can loses its fizz, becomes less palatable, less portable and less likely to be finished.

Ask patient to use straw to reduce SSB’s caries inducing potential. Using straw, the drink can be directly swallowed by minimum contact with teeth.

Diabetic parent should made aware that “genetic predisposition for type-2 DM is even more stronger than for type-1 DM”.^24,25^

Encourage coconsumption of dairy foods rich in calcium and phosphate, when ingesting acidic foods and beverages. A sports drink should not be used as mouthwash and need to be swallowed with minimum contacting teeth and, after consumption, patient should rinse mouth with water to minimize residual acidic liquid in the mouth.

The ultimate message should be recommended by dentist to the parents:

“Eat Less Sugar and Eat Sugar Less Often”.

## CONCLUSIONS AND COMMENTS

Brushing, immediately after consumption of SSB drinks should be avoided because the enamel becomes transition-ally softened. Patients should wait an hour before brushing to allow teeth to remineralize and enamel to harden.

Patients with exposed dentine should be advised to use soft toothbrush to minimize tooth abrasions. Regular checkup, fluoridated toothpaste and a specific fluoride regimen for these patients should be established.

Diet is less of a factor in caries prevention when adequate oral hygiene and daily fluoride are present. Healthy eating habits and optimal oral hygiene are important aspects to minimize potential of teeth decay.

After reviewing the literature, it can be recommended and concluded that unhealthy eating and drinking habits become part of modernization, which ultimately renders the children and young adults into medical and dental complications. Counseling for changing life-style of the families, especially high-risk group people, may help improving the general and oral health of children and adolescents.

All age consumers of SSB should be motivated by:

“Think Before You Drink”.
